# Benefits of health reform for households in rural South Africa following implementation of ward-based primary healthcare outreach teams: a qualitative inquiry

**DOI:** 10.1080/16549716.2018.1527666

**Published:** 2018-10-17

**Authors:** Landiwe Khuzwayo, Mosa Moshabela

**Affiliations:** a Discipline of Public Health Medicine, School of Nursing and Public Health, University of KwaZulu-Natal, Durban, South Africa; b Discipline of Rural Health, School of Nursing and Public Health, University of KwaZulu-Natal, Durban, South Africa; c Research Department, Africa Health Research Institute, Mtubatuba, South Africa

**Keywords:** Primary healthcare, health reform, access to healthcare, outreach teams, benefits

## Abstract

**Background**: Major national primary healthcare reforms are seldom implemented, and few studies have explored the benefits of primary healthcare outreach teams to rural households, a knowledge gap we sought to address with this study.

**Objective**: The objective of this study was to explore the community benefits in the context of PHC services delivered in rural households by outreach teams.

**Methods**: The study was conducted in the iLembe District on the east coast of KwaZulu-Natal, South Africa between July 2015 and January 2017. In-depth, explorative and semi-structured qualitative interviews were conducted as part of a mixed-method study. A total of 21 in-depth interviews with key informants and four focus group discussions (n = 28) were conducted with purposefully sampled households and outreach team members. Content analysis was used to explore and understand the households’ experiences of primary healthcare services provided by outreach teams.

**Results**: Household members benefited from outreach team services tailored to specific households and individuals, which improved the efficiency of healthcare services, access to appropriate health information and the overall experience of healthcare, particularly among those who are physically unwell, on chronic treatment, default treatment or immunisation, or who need referrals for clinical and social services. The benefits to household members included personalised care in the home, improved referral pathways, awareness of health events, improved adherence to treatment and reduction in opportunity costs of healthcare.

**Conclusion**: It is perceived that participants have benefitted from the model of PHC service delivery by outreach teams through improved access to healthcare services, and by allowing community members to receive services that are responsive to their needs since the outreach team members’ advocate and negotiate to deliver services on behalf of community members. These stated benefits, as perceived by household members, have the potential to improve health outcomes and increase satisfaction levels amongst household members.

## Background

The Alma-Ata Declaration advocated for primary healthcare (PHC) as the strategy to strengthen health systems in developing countries [1]. Countries in Asia and South America had been implementing programmes using the principles of PHC since the 1970s and 1980s, and these regions of the world have since attained significant and tangible improvements in health. Thailand implemented PHC in 1977, using village health volunteers and health communicators, while Brazil introduced a large-scale Family Health Programme that included community-based teams of physicians, nurses and community health workers (CHWs) [2]. Countries such as India, Brazil and Cambodia, through their community-based PHC team models, have reported improved health outcomes such as the reduction in child mortality as well as referral pathways [3–6]. The community-based PHC teams provide a range of services at a municipality level, such as home-based care, health promotion, rehabilitation services and treatment of some ailments. These services are extended to the household level through regular visits to households. Household users of these services report high levels of overall satisfaction with services, displayed fewer symptoms when compared to those who only had access to the physician [7,8], and felt that they had been treated with respect and courtesy [9]. The family and community models of delivering PHC services have also been shown to reduce emergency department use, improve access to healthcare, enhance patient satisfaction, and improve patient health and quality of life [10,11].

In South Africa, the healthcare system struggles to cope with the high demand for services due to a large burden of disease, and as a result, households and communities are said to be responsible for 90% of the caring of the ill, often provided at home [12,13]. In rural areas, access to healthcare is further compromised by a number of health system and socio-economic barriers [14]. Providing care in the home can reduce unmet PHC needs, and ultimately reduce health inequalities of marginalised and hard-to-reach communities [13]. In response to its national health crisis, the South African Government had developed a comprehensive plan to transform the health system of the country in order to achieve the goal of health for all. The impetus for the health reforms was created by the need for National Health Insurance as a long-term goal for financial-risk protection and universal health coverage by providing an universally accessible and equitable package of healthcare [15]. Central to this transformation was the need to strengthen PHC as the backbone of the healthcare service delivery system, and to fulfil the calls for the revitalisation of PHC by the World Health Organisation (WHO) [16]. PHC revitalisation is a key pillar of health reform in South Africa [17], with the goal of improving community-based service delivery by ‘pro-actively reaching out to families’, emphasising disease prevention, health promotion and community participation, and addressing the social determinants of health and their impact on the health of communities [18]. As a result, ward-based PHC outreach teams were established in 2011 as a mechanism to achieve universal coverage. These outreach teams were linked to PHC health facilities and leveraged facility-based resources to conduct their duties.

In South Africa, outreach teams are made up of a professional nurse who is the team leader, an environmental health officer, health promotion practitioner and four to five community health workers (CHWs). Although the literature is not specific on the frequency which outreach teams visit households, their role is to provide a range of PHC services, including health promotion, prevention and early detection of disease, ante-natal and post-natal care, and psychosocial support to communities, households and individuals [11,19]. The team leader is appointed by the health district manager, reports to the linked facility manager, and is responsible for ensuring that the work of outreach teams is appropriately distributed and aligned to service delivery targets [20]. The CHWs within the outreach teams are employed on a fixed-term contract for a year by the department of health, and report to the team leader, who supervises their work performance. If, after two years, the CHWs do not meet the job competency requirements, they are no longer eligible to continue working within the outreach teams [20]. The role of the CHWs within the outreach teams is mainly to strengthen the interface between the households and health facility services [12,13]. The CHWs provide various services, which include linking communities to the healthcare system, preventative care and keeping track with disease outbreak [21], as well as offering community-based healthcare and social support to complement rather than substitute the more specialised services of the healthcare system [13]. In a study conducted in rural South Africa, it was reported that the CHWs negotiated and established a personalised client-centred package of care, and that they continually assessed and adapted the package of services for each client and household as circumstances evolved [12]. CHWs repeatedly visits clients, personally attend to them, collect their medicines and advocate for them with either their families or health providers in cases of access barriers [13]. The WHO indicate that CHWs are essential in PHC as they have been shown to assist in improving health outcomes in communities [12,21].

Based on our previous study on the role of PHC outreach teams in South Africa, we determined that household members considered these outreach teams to be valued resources as their functions and activities brought services closer to people, mostly by providing health education, and by delivering chronic medication for clinically stable patients and making referrals to clinics [22]. The results of this previous study suggested that introducing outreach teams may play a role in lightening the burden associated with pursuing healthcare in health facilities, and the availability of community-based primary healthcare and managed referral pathways may benefit households [22]. As a result, we sought to delve deeper into this question by exploring what household members perceive as the benefits they received from the outreach teams associated with PHC services in rural communities. Currently limited studies report on this model of PHC outreach teams in South Africa, since the approach has only recently been introduced as part of the broader health reform. The objective of the study was to explore the perceived community benefits associated with receiving PHC services from the outreach teams in rural communities. This user perception study will likely provide an indication of the quality of care, and assist in informing the necessary improvements in the delivery and usage of health services by PHC outreach teams [23].

## Methods

### Study design

We conducted an explorative qualitative study as part of a larger mixed-method research project to investigate the experiences and benefits of household members regarding services provided by outreach teams in rural households. The qualitative component presented the opportunity to understand the needs, and identify the benefits, barriers [24] and outcomes of using PHC outreach teams, in ways that could further inform quantitative hypotheses and measures.

### Setting

The study was conducted in the iLembe District, located on the east coast of the KwaZulu-Natal province of South Africa. The district covers 3 269 km^2^, the smallest provincial district in KwaZulu-Natal [25], with a population size of 630 464 people [26]. The district comprises a small urban area, with the majority of the area being rural. The district’s rural and traditional areas are characterised by low educational levels, high unemployment rates, and a severe lack of basic services [27]. Service delivery backlogs are common as geographical constraints, low density and low affordability levels impede the provision of basic infrastructure [27]. The district has 74 wards; at the time of the study only three outreach teams were fully functional at municipality ward level within the district. The majority of the outreach team members, particularly CHWs, work in the community they live in, and are assigned about 200 households. The study site was selected on the backdrop of a study on quality improvement in health facilities that was being conducted by the Centre for Rural Health of the University of KwaZulu-Natal, which helped facilitate access to the communities and facilities.

### Study population and sampling strategy

Study participants were purposefully selected on the basis of their experience with the outreach team services offered in the households. We conducted 21 in-depth (IDI) interviews and four focus group discussions (FGD) (n = 28); the participants for the IDI were community representatives and outreach team members in the form of five CHWs. All other participants and all participants in the FGDs were household members. For household members, the outreach teams shared their assigned lists of households, which were used as a sampling frame for participant recruitment for the in-depth interviews (IDIs), and through which key informants were approached on the basis of their availability and willingness to participate in the study. Some community participants for focus group discussions (FGDs) were recruited by the researcher concurrent to the recruitment of participants for the IDIs. Other community participants were recruited by the outreach team members, and the rest by community development workers in their respective municipal wards. Household members were included if they had received services from the outreach teams during the 12 months prior to the interview. For outreach team members, CHWs were recruited using the CHW contact list provided by the team leader. CHWs had to have been part of the team for at least six months, and working with one of the three functional teams operating at the time of the study. In the first ward, only one team member, a CHW, consented to participate, and in the second ward, no team member agreed to be interviewed. In the final ward, only one had initially agreed to participate, but at the end of her interview, she offered to call her colleagues whom had initially refused to participate, and three additional CHWs agreed to participate. All participants were above the 18 years of age.

### Data collection

Using interview guides, IDIs and FGDs were conducted with CHWs, community representatives and household members by the researcher and a research assistant between July 2015 and January 2017. In-depth interviews with community members were conducted in participants’ homes and FGDs in community halls. Interviews with CHWs were conducted at conveniently situated community sites during working hours, but between household visits in order to minimise service disruption. Table 1 shows the data collection tools and the number of interviews conducted per participant type. Venues included the ward councillor’s office, which is considered the meeting place for the outreach team members. The interviews were conducted by the first author of this manuscript who worked with a research assistant. All interviews and group discussions were conducted in IsiZulu, which is the first language of the participants, researcher and the research assistant. Data were audio recorded, transcribed and translated into English by the first author and the research assistant.

**Table 1.** Data collection tools used and the number of interviews conducted per participant type.

	Number of participants		
	Focus Group	Key Informant Interviews	Community Health Workers
Data collection tool	Focus Group Discussion Guide	Key Informant Interview Guide	Key Informant Interview Guide
Ward 1	7	5	1
Ward 2	7	3	0
	6	-	-
Ward 3	8	8	4

Source: Authors’ own work

### Data analysis

Transcripts were stored and managed using QSR International’s NVivo software. Themes and sub-themes that describe the benefits of outreach team services were extracted for this paper. In the first instance, transcripts were read and manually coded using the content analysis technique. Data was further triangulated and compared between household members, community representatives and CHW responses to increase the depth of the analysis. The researcher generated codes, and repeated codes were developed into sub-themes and themes, which were charted and refined. Table 2 outlines the data transitions from text to sub-themes and themes that emerged during data analysis. Direct quotes from participants were used to support the findings in the presentation of results, and each participant was allocated a unique but anonymous identifier, namely participant category, area, gender, and data collection method.

**Table 2.** Examples of transitions from textual to codes, subthemes and themes.

Extracts	Codes	Sub-Theme
**Theme 1: Individually tailored services**		
‘they visit old people and those that are sick people especially chronic patient, they clean, check if there is something to eat and feed them, if maybe the person is wearing diapers they would change them, I am talking about something that I have seen.’	Caring for the aged	Care and support in the home
‘there is this old man who was amputated and couldn’t walk, I use to clean his wound, cook for him and collect bandages at the clinic using my money, and he is fine now.’	Provision of HBC	
‘…it depends on the nature of your condition, because before they go they must understand the nature of your condition so that they can ask guidance from their supervisors about your condition, they are very patient.’	Tailor-made service provision	Personalised and individualised care
‘their visit depends on the household, in terms of how much problems does the household have, if they need to do follow ups they would then come back for follow up to monitor that cases,’	Focused services	
**Theme 2: Optimising efficiencies in healthcare services**		
‘I would skip a month and not take the child for immunisation. They [outreach team] came and checked the immunisation card, and assessed what immunisation doses has the child missed, they would then weigh and give missed immunisation doses to the child.’’	Attending to missed opportunities	Minimising missed opportunities
‘his mother left him here terminally ill, I could literally see his ribs. I contacted our CHW; she assisted us to get the child started on treatment (ARV) and also helped us get food vouchers. She was very supportive throughout the process, more especially in the beginning when the child vomited after taking the pills.’	Linkage	Strengthening of referral pathways
‘if your problem requires that you go to the clinic they do write it a referral form for you and you take it with you to the clinic’	Referral	
**Theme 3: Appropriate health information**		
‘….as I am talking to you, I am waiting for the nurses to come to the crèche for immunisation campaign. Our CHW told us they will be coming today. I need to take my grandchildren [to the crèche] with their immunisation cards.’	access to information	Awareness of events and services
‘we have learnt a lot of things that we didn’t know especially as we are lazy to go to the clinic when they come in the community we are able to ask them things we don’t know and they would teach us,’	Learning opportunity	
‘I have boy children, they came and told me that I must send the boys for circumcision at the clinic, I sent my boys and they were circumcised and they also told me that we must go for cervical screening, I am going on Saturday.’	First-hand information	
‘they come here to check if we are ok, if you have a problem, they counsel you and tell you how you are going to get help, they also came to bring me the wheelchair, I am left alone during the day, and I can’t make food because I fall on crushes but now with the wheelchair I can make food and eat.	Independency	Patient Motivation and Empowerment
‘this team is helping. There are people who don’t like taking their pills, especially the TB and HIV treatment, as people don’t like taking pills, one becomes motivated to take their pills as they (outreach teams) constantly visit you.’	Change of mind set	
**Theme 4: Improved experience of healthcare**		
‘…like collection of medication, our taxis are very expensive, and you find out that you don’t even have the R7, and you must have R14, and it goes up again.’	Reduced out of pocket costs	Reduction of opportunity costs
‘there is no need to catch a taxi to the clinic unless they are the ones that are sending you to the clinic’		
‘the mobile clinic use not to cover certain areas that I cover and I spoke to the mobile clinic team to cover those areas and now they do come.’	Negotiation for services provision	
‘I have blood pressure, I use to drink the pills anyhow they came and taught me how I should take my treatment in order to control my condition, I started doing what they told me, I no longer have that problem.’	Importance of treatment adherence	

## Results

A total of 49 participants were interviewed, 46 of whom were females, with the average age of participants being 42 years of age. All household respondents had received household visits from, and engaged with, the outreach teams. These households hosted outreach teams for an average duration of 30 to 60 minutes, largely dependent on the nature and number of issues being addressed. The benefits to the households from these outreach team visits are presented hereunder as themes and subthemes.

### Themes and subthemes

Four categories of benefits emerged during analysis: (1) individually tailored services, (2) optimising efficiencies in healthcare services, (3) appropriate health information and (4) improved experience of healthcare. The themes and sub-themes are presented in Table 3, and further explored below.

**Table 3.** Themes and subthemes.

Theme	Sub-theme
Individually tailored services	Care and support in the home
	Personalised and individualised care
Optimising efficiencies in healthcare services	Minimising missed opportunities
	Strengthening of referral pathways
Appropriate health information	Awareness of events and services
	Patient motivation and empowerment
Improved experience of healthcare	Reduction of opportunity costs
	Improvement in treatment adherence

Source: Authors’ own work

### Individually tailored household services

Household members benefited from individually tailored outreach team services in the form of care and support in the home, by necessity tailored to household needs, and personalised care delivered according to unique individual needs.

#### Care and support in the home

Household members who could not care for themselves were reported to receive home-based care services from the outreach teams, including services such as house cleaning, patient feeding and diaper changing, most recognisable among the elderly people and patients who were ill and thus unable to leave their homes.

*They visit old people and those that are sick, especially chronic patient. They clean, check if there is something to eat and feed them. If maybe the person is wearing diapers, they would change them. I am talking about something that I have seen.’* FGD, Isithebe, female, community member

#### Personalised and individualised care

In addition to the regular and standard checks when visiting households, household members appreciated outreach team services that were tailored based on the perceived needs of the individual household members. Solutions were therefore adapted to suit the circumstances of each household and individual visit by outreach team members.

*…it depends on the nature of your condition. Because before they go, they must understand the nature of your condition so that they can ask guidance from their supervisors about your condition, they are very patient.’* IDI, Sadloko, female, community member

However, such home-based, tailored and personalised care services appeared to carry personal costs for some outreach team members.

*There is this old man who was amputated and couldn’t walk. I used to clean his wound, cook for him and collect bandages at the clinic using my money, and he is fine now.’* IDI, Groutville, female, outreach team member, community care worker 2

### Optimising efficiencies in healthcare services

Additional benefits for household members were improved access to healthcare services, where missed opportunities were minimised since the introduction of outreach teams, and strengthened referral pathways, as they were able to be linked appropriately to other healthcare and community resources.

#### Minimising missed opportunities

Household members reported that outreach teams were able to identify missed opportunities and fix such problems at a community level without referring them to the clinic. Furthermore, household members reported that the outreach teams examined immunisation cards (road to health cards), maternal case records to check if pregnant women were attending antenatal classes according to the schedule, and chronic patient carrier cards to monitor if chronic patients are adhering to treatment. In some cases they will do a pill count. Community members were, as a result, able to witness the role of outreach teams in minimising the missed opportunities for households.

*I would skip a month and not take the child for immunisation. They [outreach team] came and checked the immunisation card, and assessed what immunisation doses has the child missed, they would then weigh and give missed immunisation doses to the child.’* IDI, Sans Souci, male, community member

#### Strengthening of referral pathways

The outreach teams wrote referral letters for household members even though they did not get any formal report from other institutions. One participant mentioned how she was linked with the Department of Home Affairs to get birth certificates for the children of her late sister, and was also able to apply for a child grant from the Department of Social Development. Moreover, participants in focus group discussion mentioned that the outreach teams referred children to the clinic for deworming and other immunisation services. Another participant mentioned how her grandson survived after she spoke to the CHW.

*His mother left him here terminally ill. I could literally see his ribs. I contacted our CHW. She assisted us to get the child started on treatment (ARV), and also helped us get food vouchers. She was very supportive throughout the process, more especially in the beginning when the child vomited after taking the pills.’* IDI, Isithebe, female, community member

### Appropriate health information

Households reported improved access to health information, and felt empowered and motivated to observe procedures necessary to maintain their health and wellbeing.

#### Awareness of health events and services

The outreach team used household visits to inform household members of new health information, planned campaigns or newly introduced services. The CHWs reported that the health information given to the households depended on household needs or situations. Outreach teams reminded household members of special activities, such as circumcision, immunisation campaigns and cancer screening that would be conducted in the facility and community on specified days. The community was therefore able to use such services, and collectively benefit from the up-to-date information they received.

*… .as I am talking to you, I am waiting for the nurses to come to the crèche for immunisation campaign. Our CHW told us they will be coming today. I need to take my grandchildren [to the crèche] with their immunisation cards.’ IDI, Isithebe, female, community member*

#### Patient motivation and empowerment

By receiving health information and direct support from outreach teams, household members felt increasingly more empowered to care for themselves, and motivated to adhere to treatment. One participant mentioned that since she received a wheelchair from the outreach team, she was able to make food on her own and move around the house easily. The teams were perceived to help household members overcome challenges impeding access and adherence to treatment.

*This team is helping. There are people who don’t like taking their pills, especially the TB and HIV treatment, as people don’t like taking pills, one becomes motivated to take their pills as they (outreach teams) constantly visit you.’* IDI, Groutville, female, community member

### Improved experience of healthcare

Health information included benefits of treatment adherence and the risks associated with treatment avoidance. Tailor-made interventions were used to help household members receive and adhere to chronic treatment.

#### Improvement in adherence to treatment

Household members were able to discuss the difficulties in adhering to treatments and negotiate possible solutions with the outreach team. In this way, the outreach team was able to propose solutions that were responsive to individual needs, with the potential to maximise treatment benefits. Outreach teams further assisted with psychosocial interventions, such as disclosure of HIV status. They also made arrangements with health services to accommodate the socio-economic circumstances of their patients, in order to maximise their adherence to treatment.

*There was one gentleman who used to default on both TB and antiretroviral treatment. He was a contract worker, and would go with the contractors for two months and not collect his medication. I advised him that before he goes, he must inform the nurse at the clinic and request for two months treatment, he did that and now he is fine.’* IDI, Groutville, female, outreach team member, community care work

#### Reduction of opportunity costs

Some household members, particularly those who could not afford to visit the clinic, reported a reduction in out-of-pocket expenditure on healthcare, as outreach team members were able to bring chronic medication to a patient’s home, instead of the patient having to visit the clinic. In this way, the outreach team members helped to save household members money and reduce the strain on their finances.

*…like collection of medication, our taxis are very expensive, and you find out that you don’t even have the R7, and you must have R14, and it goes up again.’* IDI, Sadloko, female, community member

Community members valued the beneficial increase in access to healthcare services resulting from the direct intervention of outreach teams. In two communities, a new mobile clinic service was introduced following the needs assessment and lobbying done by the outreach teams.

*The mobile clinic used to not cover certain areas that I visit, and I spoke to the mobile clinic team to cover those areas, and now they do come.’* KII, Groutville, female, outreach team member, community care work 3

## Discussion

In this study, we sought to explore household benefits through PHC outreach teams implemented at municipality ward level as part of the on-going healthcare reform in South Africa. Household members benefited from individually tailored outreach team services, perceived improved access to healthcare services, suitable health information and perceived overall improvements in the experience of healthcare. These benefits, as perceived by household members, have the potential to improve health outcomes and increase satisfaction levels amongst household members [28].

The study revealed that the provision of care and support by outreach teams were conducted mainly in the form of household-level care, which reduced the need for clinic referrals and frequent clinic visits. Providing care in the home has the potential to improve unmet PHC needs, and ultimately reduces health disparities of marginalised and hard-to-reach rural communities [13,22]. The WHO emphasises that one of the most effective ways of ‘closing the equity gap’ within a population is to address the health and healthcare needs of those most disadvantaged [29]. The results also show that household members benefit from services that are tailored and personalised to their health needs as they find it easier to understand and manage their conditions with the support of the CHWs. Receiving these services could lead to changes in health literacy, improved self-management and better health outcomes. Individually tailored, personalised care was reported as a benefit since it enabled household members to discuss treatment adherence problems and probable solutions with outreach teams.

The outreach teams offered solutions that were responsive to individual needs and made arrangements with health services to accommodate the socio-economic circumstances of household members in order to maximise treatment adherence. However, the outreach team members sometimes used their own resources to provide these services and assist households. This practise has been reported in other studies where CHWs donated personal resources in order for their patients to maintain their treatment and the relationships they have established with the households [13]. Opportunity costs, mainly transport costs, are common barriers to healthcare access, and often lead to missed appointments, delayed or missed health care [30]. Evidence from other studies show that once PHC is not accessible or effective, people delay seeking help, rely on emergency care, and lose the benefits of continuity of care [29]. Furthermore, outreach team services were perceived to close the gap in primary healthcare delivery by addressing and responding to health needs at household level, thereby overcoming barriers to access such as high healthcare-related expenditure often experienced by these rural communities.

Household members in this study reported to have benefited from the health information and direct support provided by outreach teams, resulting in feelings of motivation and empowerment to care for their health conditions and take their medication as prescribed. Healthcare providers can facilitate these forms of patient empowerment and motivation benefits if they implement a patient-centred approach of care within PHC reforms that recognise patients’ experiences, priorities and preferences [31]. Household members in this study valued and appreciated the benefits of strengthened referral pathways brought about by the outreach team services. However, previous research showed that most referrals occurred largely because outreach teams offered a limited package of services that should be expanded, which may in itself put into question the scope of work appropriate for outreach teams within the rural South African context [22]. Similar to previous studies, results from this study suggest that there is no feedback mechanism in place between the service providers and the outreach teams. Established referral procedures and feedback mechanisms between home-based care and the health care system [13] are necessary aspects of PHC reforms. Studies conducted on treatment adherence show that a good healthcare worker-patient relationship can contribute to better treatment adherence [32–34]. Furthermore, literature shows that patients provided with adequate knowledge about treatment show high adherence rates as patient behaviour is largely influenced by knowledge and acceptance of their disease and treatment [33,34].

## Conclusion

Community members perceived the services provided by outreach teams to be responsive to households’ needs at a reduced expense to the patients. The model of PHC service delivery by outreach teams allows community members to enjoy the benefits of individually tailored, personalised care, which offers an opportunity for community members and outreach teams to discuss and negotiate possible solutions to treatment adherence and disease management challenges, as well as other challenges faced by households. Due to the shortage of patients’ resources, outreach teams use their personal resources to promote success of these services. The outreach teams have the potential to be effective in meeting community needs and ultimately contribute to the success of health reforms.

## Limitations

At the time of the study three out of 74 wards had outreach teams, and as result, our study included only those wards with functional outreach teams. Therefore, future studies should attempt to include more municipal wards and geographic locations beyond rural KwaZulu-Natal, South Africa. Interviews were limited to individuals purposely selected by the outreach teams, as the researcher selected participants based on the list of suggested individuals. This could have introduced some degree of bias through inclusion of individuals with established relationships to outreach team members. However, the purpose of the study necessitated participation of households with a relatively richer experience with outreach teams. Furthermore, the details of individuals selected from the lists of households were not shared with the outreach teams in order to minimise bias. At the time of the study the outreach teams consisted of professional nurses and CHWs, but only CHWs agreed to be interviewed given their knowledge of households, thus omitting other key members of outreach teams such as the team leader. Future studies need to be conducted once all categories of staff are employed in order to capture their views and experiences.

## Paper context

In South Africa the ward-based outreach teams were established as part of the re-engineering of primary health care as a mechanism to achieve universal coverage. Their role is to provide basic primary healthcare services. Community members benefit from outreach team services as their services are responsive to household needs at a reduced expense. These teams should be adequately resourced to be effective and contribute to the success of health reforms.
